# Improved epidermal barrier formation in human skin models by chitosan modulated dermal matrices

**DOI:** 10.1371/journal.pone.0174478

**Published:** 2017-03-23

**Authors:** Arnout Mieremet, Marion Rietveld, Samira Absalah, Jeroen van Smeden, Joke A. Bouwstra, Abdoelwaheb El Ghalbzouri

**Affiliations:** 1 Department of Dermatology, Leiden University Medical Centre, Leiden, the Netherlands; 2 Division of Drug Delivery Technology, Leiden Academic Center for Drug Research, Leiden University, Leiden, the Netherlands; NYU Langone Medical Center, UNITED STATES

## Abstract

Full thickness human skin models (FTMs) contain an epidermal and a dermal equivalent. The latter is composed of a collagen dermal matrix which harbours fibroblasts. Current epidermal barrier properties of FTMs do not fully resemble that of native human skin (NHS), which makes these human skin models less suitable for barrier related studies. To further enhance the resemblance of NHS for epidermal morphogenesis and barrier formation, we modulated the collagen dermal matrix with the biocompatible polymer chitosan. Herein, we report that these collagen-chitosan FTMs (CC-FTMs) possess a well-organized epidermis and maintain both the early and late differentiation programs as in FTMs. Distinctively, the epidermal cell activation is reduced in CC-FTMs to levels observed in NHS. Dermal-epidermal interactions are functional in both FTM types, based on the formation of the basement membrane. Evaluation of the barrier structure by the organization of the extracellular lipid matrix of the stratum corneum revealed an elongated repeat distance of the long periodicity phase. The ceramide composition exhibited a higher resemblance of the NHS, based on the carbon chain-length distribution and subclass profile. The inside-out barrier functionality indicated by the transepidermal water loss is significantly improved in the CC-FTMs. The expression of epidermal barrier lipid processing enzymes is marginally affected, although more restricted to a single granular layer. The novel CC-FTM resembles the NHS more closely, which makes them a promising tool for epidermal barrier related studies.

## Introduction

Human skin equivalents (HSEs) are *in vitro* developed three dimensional models of the human skin, resembling many characteristics of the native human skin (NHS). Applications of HSEs include basic research about skin biology, skin diseases, determination of the toxicological profile of novel molecular entities and HSEs are reliable alternatives for animal testing [1]. However, the barrier functionality of current HSEs deviates from NHS, which potentially causes ambiguity for the *in vitro-in vivo* correlation. To improve the applicability of HSEs, these should mimic the barrier formation and organization of NHS more closely.

The permeability barrier function of the human skin relies mainly on the outermost layer of the epidermis, in which the corneocytes are embedded in a lipid matrix, known as the stratum corneum (SC). The lipid matrix in the SC is the only continuous pathway for compound penetration and is therefore important in the functionality of the skin barrier. Major constituents of the lipid matrix are ceramides (CERs), free fatty acids (FFAs) and cholesterol. The CERs consist of a large number of subclasses, based on the type of sphingoid base coupled to a specific fatty acid chain [2]. Within the SC, the FFA and CER subclasses span a broad characteristic range of entities, based on the carbon chain length and head group architecture [3, 4]. Together, the SC lipids are highly organized and form crystalline lamellar phases. Both a short periodicity phase (SPP) with a repeat distance of around 6 nm and a long periodicity phase (LPP) with a repeat distance of around 13 nm are present in the SC of NHS [5, 6].

When comparing the SC lipid matrix of HSEs to that of NHS, many similarities are observed. Amongst others, the presence of the twelve well-known CER subclasses is highly similar, which are detected in NHS as well as in the HSEs [7]. The differences include the increased level of mono unsaturated FFAs, reduced carbon chain lengths of CERs and FFAs and an altered CER subclass profile [7, 8]. Consequently, the SPP is not formed, the LPP repeat distance is reduced and the lateral packing of the lipids is less dense (hexagonal) compared to NHS (primarily orthorhombic) [8]. According to our hypothesis, these deviations in the lipid matrix reduce the permeability barrier in HSEs, including increased transepidermal water loss (TEWL) [8, 9]. Furthermore, in HSEs the epidermal homeostasis is altered, based on the expression of the hyper-proliferative associated marker keratin 16 (K16), the altered localization of terminal differentiation markers [8, 10, 11] and several lipid processing enzymes [7].

A widely used type of HSE is the full thickness human skin model (FTM), which consists of primary fibroblasts in the dermal equivalent and primary keratinocytes in the epidermal equivalent. Incorporation of both dermis and epidermis resembles the NHS tissue architecture to a high extent. In FTMs, the fibroblast-keratinocyte cross talk is essential for epidermal morphogenesis and formation of the basement membrane (BM) [12]. This BM directs the polarity, proliferation, differentiation and migration of the keratinocytes.

Several types of dermal equivalents are described, where the dermal matrix ranges from artificial scaffolds [13, 14], fibroblast-derived dermal matrix [15] to de-epidermised or de-cellularised dermis [16, 17]. Each offers its advantages and disadvantages, as each dermal matrix affects the attachment, morphology and activity of the incorporated fibroblasts. Due to the dermal-epidermal cross talk, the dermal matrix could have a high impact on the epidermal homeostasis and barrier formation [18, 19]. Therefore, modulation of the dermal matrix offers an interesting method to enhance the barrier formation in HSEs.

The aim of this study is to advance the resemblance of native skin architecture and barrier properties in FTMs by modulating the dermal matrix with the biopolymer chitosan. For this purpose, the dermal matrix of the FTM is modulated with the biopolymer chitosan to enhance the functionality of the dermal matrix. This approach resulted in the development of the collagen-chitosan full thickness human skin model (CC-FTM). Chitosan is biodegradable, easily available and serves as an excellent substrate for cell adhesion [20]. Ionic interactions between the amine groups of chitosan and the carboxyl groups of the collagen will form a stabilized dermal matrix during preparation [21–23]. In addition, during CC-FTM development, cationic sites on the chains of chitosan are still present and could benefit cellular adhesion [24]. The biocompatibility of chitosan in HSEs has been demonstrated by the reported incorporation of chitosan in a sponge-like dermal matrix [25–27]. Incorporation of chitosan in the dermal matrix potentially enhances the functionality of the dermal and epidermal equivalent and therefore could be advantageous for the overall homeostasis and ultimately the barrier formation.

In this study, evaluations between the novel CC-FTM, the established FTM and NHS has been performed regarding epidermal morphology, proliferation, differentiation programs, BM formation and barrier lipid processing enzymes. Detailed analyses of the SC lipid characteristics and barrier functionality revealed improved epidermal barrier formation in the novel FTM type.

## Materials and methods

### Primary cell isolation and culture

Isolation of primary human fibroblasts and keratinocytes was performed from surplus human breast skin [15]. Declaration of Helsinki principles are followed with obtainment of primary human skin cells from healthy donors, exactly as stated and described before [28]. Once fat tissue was removed, the dermis and epidermis were separated after overnight incubation of the skin in 2.4 U/mL dispase II *(*Roche, Almere, The Netherlands). Primary keratinocytes were isolated from the epidermis after treatment with 0.05% (w/v) trypsin (BD Falcon, Breda, The Netherlands) and cultured as described before [15]. Primary fibroblasts were isolated after incubation of the dermis in a 3:1 (w/w) mixture of collagenase (Gibco) and dispase II (Roche) for two hours at 37°C and cultured as described before [15, 29]. All isolated primary cells were tested and found negative for mycoplasma contamination.

### Dermal equivalent preparation

#### Fibroblast-populated collagen matrix

Development of dermal equivalents was performed as described earlier [8]. The hydrated collagen (4 mg/mL) was isolated from rat-tail tendons. Obtainment of rat-tail tendons occurred as described before, including the ethics statement [28]. Below a 1 mL cell-free collagen mixture with on top a 3 mL fibroblasts containing mixture (1.2–1.5 x 10^5^ fibroblasts in each dermal equivalent) were produced onto filter inserts (Corning Transwell cell culture inserts, membrane diameter 24 mm, pore size 3 μm; Corning Life Sciences, The Netherlands) and allowed to polymerize at 37°C. After polymerization, the dermal equivalents were cultured as described elsewhere [8, 29] with fresh supplementation of 45 μM vitamin C (Sigma).

#### Fibroblast-populated collagen-chitosan matrix

Chitosan (cat. no. 448877, Sigma) was dissolved in 0.1% acetic acid at 4°C. Based on pilot studies, a final chitosan concentration of 5 mg/mL was used. The collagen-chitosan dermal equivalent was generated in a single step by mixing the 4 mg/mL collagen solution with the 5 mg/mL chitosan solution in a 3:1 (v/v) ratio, HBSS, 1M NaOH and a fibroblast containing FBS solution. Four mL of this mixture (equals 1.3–1.6 x 10^5^ cells fibroblasts each dermal equivalent) was allowed to polymerize at 37°C. Subsequently, equal procedures were followed as the fibroblast-populated collagen matrix.

### Generation of FTMs

Primary keratinocytes (2.5x10^5^/model) in their first passage were seeded onto each dermal equivalent as described before [8, 29]. The FTMs were kept submerged for total of four days. Hereafter, the FTMs were lifted to the air-liquid interface and developed for 14–17 days [30].

### Tissue imaging

#### Fixation of the tissue

Cross-sections of each FTM were either snap frozen for cryofixation or fixated with formaldehyde for paraffin embedding. For cryofixation, the tissue was placed in a gelatine capsule containing Tissue-Tek^®^ O.C.T.^™^ Compound (Sakura Finetek Europe B.V., Alphen aan den Rijn, The Netherlands) and snap frozen in liquid nitrogen. For paraffin embedding, the tissue was fixed in 4% (w/v) formaldehyde (Added Pharma, Oss, The Netherlands), dehydrated and embedded in paraffin.

#### Immunohistochemical analyses

General morphological analysis was performed with 5 μm paraffin embedded tissue sections through staining with haematoxylin and eosin (HE; Klinipath, Duiven, The Netherlands). Immunohistochemical analyses of Ki67, keratin 10 and 16 were performed on 5 μm paraffin embedded tissue sections. After deparaffinization and rehydration, heat-mediated antigen retrieval in citrate buffer was performed, followed by blocking with 2% normal human serum (Sanquin, Leiden, The Netherlands).

Stainings were performed using the streptavidin-biotin-peroxidase system (GE Healthcare, Buckinghamshire, United Kingdom), according to the manufacturer’s instructions. Stainings were visualized with 3-amino-9-ethylcarbazole (AEC), counterstained with haematoxylin and sealed with Kaiser's glycerine. Specifications of the primary and secondary antibodies are provided in S1 Table. Visualization of the sections occurred using a light microscope (Zeiss Axioplan 2, Zeiss, The Netherlands).

#### Immunofluorescence analyses

Immunofluorescence staining of loricrin, collagen type IV, β-glucocerebrosidase (GBA), acid sphingomyelinase (aSMase), ceramide synthase 3 (CER-S3) and elongation of very long chain fatty acids protein 1 (ELOVL1) was performed with 5 μm formalin-fixed paraffin embedded sections. After deparaffinization and rehydration, heat-mediated antigen retrieval in citrate buffer was performed and followed by blocking with 2% normal human serum (Sanquin). During the collagen type IV staining, a protease treatment using a 0.025% protease solution (Sigma) was performed before incubation with the primary antibody. The sections were incubated with the primary antibody overnight at 4°C directly after blocking. After phosphate-buffered saline (PBS) washout secondary antibody was applied for 1 hr at RT and subsequently the sections were mounted with Vectashield containing DAPI for visualization of the nuclei (Vector Laboratories, The Netherlands). Immunofluorescence stainings of vimentin and laminin 332 were performed on 5 μm thick frozen sections, which were dried overnight and fixed in acetone for ten minutes. Primary and secondary antibody application is equal as paraffin embedded sections as well as mounting. Visualization of the sections occurred using a fluorescence microscope (Leica CTR5000, Leica, The Netherlands).

### Determination of the epidermal thickness and proliferation index

The epidermal thickness was determined through quantification of eight images of each FTM type of various tissue regions after HE staining with 200x magnification. The outline of the epidermal area was determined with Adobe Photoshop (CS6 version 13.0) and the number of pixels in the epidermal area was measured. This was transformed to the area in squared micrometres. The proliferation index (PI) was determined by counting the number of Ki67 positive nuclei among the total number of cells in the basal layer. A minimum of 100 basal cells were counted at three different regions of each section. The resulting proliferation index is the percentage of positive stained nuclei. For both estimations, the data is presented as the mean of four independent experiments ±SD.

### Counting stratum corneum layers

Cryofixated sections were sliced 5 μm using a cryotome (Leica CM3050S), dried overnight and fixed in acetone for ten minutes. Sections were stained for one minute with a 1% (w/v) safranin O (Sigma) solution dissolved in Millipore water. After water washout, a 2% (w/v) KOH solution was applied on the sections for 25 minutes to swell the corneocytes. After removal of the KOH solution, the glass slides were washed with Millipore water and enclosed with Kaiser’s glycerine. Images were obtained with 400x magnification.

### Stratum corneum isolation

The SC of FTMs was isolated through overnight incubation with a 0.1% (w/v) trypsin/PBS solution at 4°C followed by incubation at 37°C for 1 hour. The SC was isolated and washed with 0.1% trypsin inhibitor and Millipore water. The SC was air-dried and stored under Argon gas over silica until further use.

### Small angle X-ray diffraction analysis

Small-angle x-ray diffraction (SAXD) measurements were performed at the European Synchrotron Radiation Facility (ESRF, Grenoble, France) at station BM26B as described in detail elsewhere [31]. The SC was hydrated over a 27% (w/v) NaBr solution during 24h at room temperature. SAXD patterns were detected with a Frelon 2000 charge-coupled device (CCD) detector at room temperature for a period of 2 x 150 seconds. The scattering intensity (I) was measured as a function of the scattering vector q (in reciprocal nm). The latter is defined as q = (4πsinθ) /λ, in which θ is the scattering angle and λ is the wavelength. From the positions of a series of equidistant peaks (located at q_n_), the periodicity, or d-spacing, of a lamellar phase was calculated using the equation q_n_ = 2nπ/d, n being the order number of the diffraction peak [5]. The repeat distance of the lamellar phases was calculated based on the first, second and third order diffraction peak of the lamellar phase.

### Lipid extraction and LC-MS analysis

Extraction of total SC lipids was performed based on an adjusted Bligh and Dyer method as described by Boiten *et al*. [32]. To determine the weight percentage of lipids in the SC, dry SC weight was measured before and after extraction. Nomenclature of the CER subclasses is followed according to Motta *et al*. [2] and listed (S1 Fig) [33, 34]. To analyse the CER subclasses and chain length distribution in each of the subclasses, the organic phase was collected and evaporated under a stream of nitrogen at 40°C. The obtained lipids were dissolved in a suitable volume of heptane:chloroform:methanol (95:2.5:2.5 v:v:v). The lipids were analysed by liquid chromatography—mass spectrometry (LC-MS) according to the method described elsewhere ([35] modified by Boiten *et al*. [32]. Briefly, CERs were separated and detected with an Acquity UPLC H-class (Waters, Milford, MA, USA) coupled to an XEVO TQ-S mass spectrometer (Waters, Milford, MA, USA). Separation of CERs occurred in normal phase on a PVA-Sil column (5μM particles, 100x2.1mm i.d.)(YMC, Kyoto, Japan) using a gradient starting with 98% heptane towards 50% (Hep:IPA:EtOH, 50:25:25, v:v:v) [35]. A flow rate of 0.8 mL/min was used. Detection was performed using APCI in positive ion mode, measuring in full scan from mass 350–1200 between 1.25–12.5 min. The total lipid concentration of all samples was around 0.3 mg/mL and the injection volume was set to 5 μL. Relative abundance of the FTMs and CC-FTMs was determined through quantification of the AUCs followed by a correction using a single internal standard as in Boiten *et al*. [32]. Relative abundance of benchmark values for NHS was determined based on quantification of the AUCs of a mixture of lipid extracts from eight different donors.

### Transepidermal water loss measurements

Isolated SC was placed in a flow-through diffusion cells (PermeGear, Inc, Bethlehem, USA) filled with Millipore water with tubes connected to prevent the diffusion cell to leak or dry out. After a 30 minute incubation period, the closed chamber evaporimeter (Aqua Flux AF200: Biox Systems Ltd, London, UK) [36] was placed on top of the SC for 15 minutes. Next, transepidermal water loss (TEWL) measurements were performed after every 20 seconds during a time period of 900 seconds.

### Ethics statement

This ethics statement is identical to that of Haisma *et al*. [28]. All primary human skin cells from healthy donors used by the Department of Dermatology are isolated from surplus tissue collected according to article 467 of the Dutch Law on Medical Treatment Agreement and the Code for proper Use of Human Tissue of the Dutch Federation of Biomedical Scientific Societies. According to article 467 surplus tissue can be used if no objection is made by the patient. This means that the patient who will undergo plastic surgery is well informed on the research. In case he/she refuses the patient has to sign the inform consent form, if they agree they do not sign. This approach differs from other countries. None of the authors were involved in the tissue sampling and only birth date, gender and skin type of the subjects was known. The Declaration of Helsinki principles were followed when working with human tissue.

The rat-tails were obtained from rats of the Animal Facility (Proef Dier Centrum) of the Leiden University Medical Center, The Netherlands. Rats came from the breeding facility, or were control animals of other studies and died by CO2 asphyxiation. Because no living animals were used for the purpose of this study, no approval was needed from the Institutional Animal Care and Use Committee. This is in agreement with the European directive for the protection of animals used for scientific purposes (2010/63/EU).

### Statistics

Statistical analyses are conducted using GraphPad Prism version 6.00 for Windows (GraphPad Software, La Jolla California USA). In general, statistical significance was determined using a paired Student’s t-tests or one-way ANOVA. The CER carbon chain length distribution, CER subclass profile and TEWL were statistically tested using two-way ANOVA with multiple comparison Holm-Šídák post-test. Statistical differences are noted as *, ** or ***, corresponding to P<0.05, <0.01, <0.001.

## Results

### Characterization of the epidermal homeostasis

**Fig 1:**
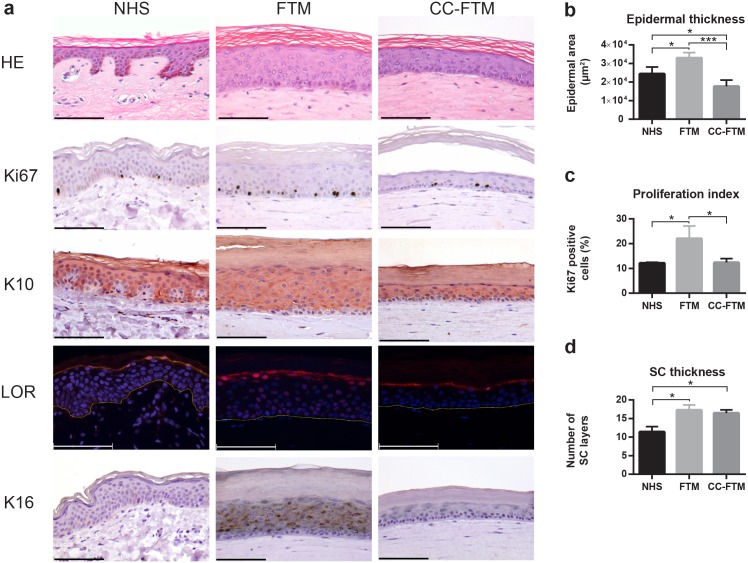
Epidermal morphogenesis in NHS, FTMs and CC-FTMS. (a) Cross-sections are examined for general morphology (HE), proliferation (Ki67), early (K10) and late (LOR) differentiation and epidermal cell activation (K16) using immunohistochemistry or immunofluorescence. The expression of loricrin (LOR) is depicted in red and the nuclei in blue, whereas the yellow dotted line indicates the dermal-epidermal junction. (b) Quantification of the epidermal thickness based on the HE staining. (c) Basal cell proliferation quantified based on the Ki67 positive cells in multiple regions of the epidermis. (d) Safranin red staining was quantified by counting the amount of corneocyte layers in the SC. Scale bar represents 100μm. *indicates p<0.05. Data represents mean ±SD of four independent experiments or skin donors.

To evaluate the effect of the modulated dermal matrix on epidermal organization and morphogenesis, full thickness human skin models with solely collagen as a dermal matrix (FTMs) and FTMs with the collagen-chitosan dermal matrix (CC-FTMs) are generated. After keratinocyte seeding onto the dermal matrices, fully developed into a stratified epidermis, the epidermal morphology is evaluated and compared to NHS. Both dermal matrices enabled the formation of a well-organized epidermis, since the presence of all four epidermal strata (including stratum basale, stratum spinosum, stratum granulosum and stratum corneum (SC)) is observed (Fig 1A). However, the addition of chitosan resulted in a decreased epidermal thickness (Fig 1B), independent of the different fibroblast and keratinocyte donors used.

Next, basal cell proliferation (Ki67), early (keratin 10; K10) and late (loricrin; LOR) differentiation and the activation of the epidermis (K16) were examined, together providing information on epidermal homeostasis. Compared to FTM the basal cell proliferation is reduced in CC-FTMs, based on the Ki67-proliferation index (PI) (Fig 1C), with a PI of 22.00 ±5.14 versus a PI of 12.33 ±1.67, respectively. The proliferation index of the CC-FTM became equal to that of NHS. The equal suprabasal expression of K10 in FTMs and CC-FTMs indicates an unaltered execution of the early differentiation program. The expression of the late differentiation marker LOR is more restricted to a single granular layer in CC-FTMs (Fig 1A), more similar to NHS. A clear difference is observed when focusing on the epidermal cell activation. The expression of K16 in CC-FTMs is strongly reduced compared to FTMs and matches expression levels as in NHS. Finally, the number of corneocyte layers in the SC was determined based on the safranin staining. In both FTM types, an equal number of corneocyte layers in the SC is observed (Fig 1D), which is increased as compared to NHS.

### Basement membrane formation and fibroblast distribution

**Fig 2:**
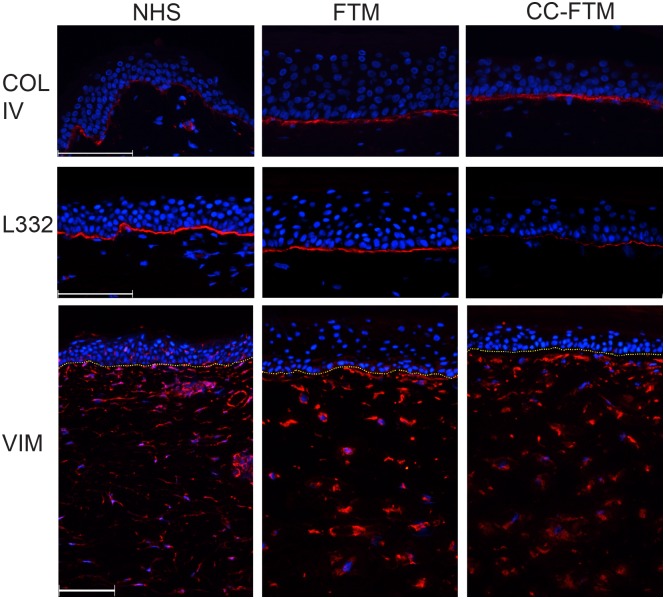
The dermal-epidermal linkage and fibroblast distribution. Cross-sections of NHS, FTMs and CC-FTMs are analysed by immunofluorescence. Collagen type IV (COL IV) is similarly expressed at the BM in both FTMs and CC-FTMs, while laminin 332 shows a delayed expression in CC-FTMs. Similar fibroblast distribution throughout the dermis is shown by vimentin (VIM). Proteins are visualized in red, nuclei are stained blue using DAPI and the yellow dotted line indicates the dermal-epidermal junction. Scale bar: 100 μm. Representative pictures are shown of four independent experiments.

Next, we examined whether the modulated dermal matrix affects the basement membrane (BM) formation. For this purpose the deposition of two BM proteins with distinct functions is examined by immunohistochemistry (Fig 2). Collagen type IV, a major lamina densa structural protein, is continuously expressed at the dermal-epidermal junction in NHS and both FTM types. In contrast, the deposition of a key component of the lamina lucida/lamina densa interface, laminin 332 (L332) is reduced in CC-FTMs. To evaluate if this was a result of an altered fibroblast distribution in the chitosan-supplemented dermal matrix or a characteristic of the CC-FTMs, we examined the fibroblasts distribution using the fibroblast cytoskeleton protein vimentin (VIM). As shown in Fig 2, VIM, is detected throughout the dermal matrices in NHS and both models, indicating a heterogeneous distribution of fibroblasts (Fig 2).

### Lamellar organization in the extracellular matrix of the stratum corneum

**Fig 3:**
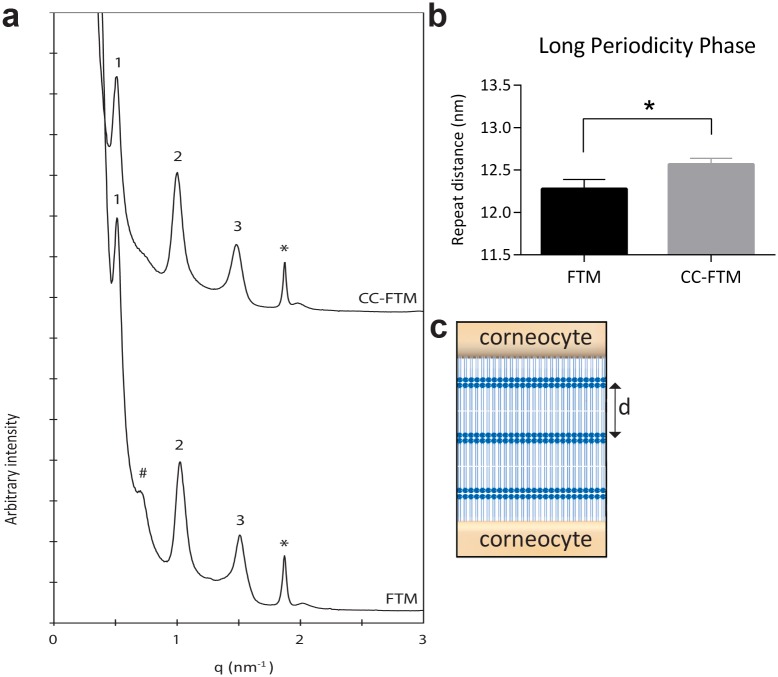
Lamellar organization of the extracellular lipid matrix in the Stratum Corneum (SC). (a) Representative diffraction pattern of the lipids in the SC of FTMs and CC-FTMs. The first, second and third order of diffraction are detected, as well as diffraction peaks attributed to separate crystalline cholesterol, indicated by the asterisk (*). In FTMs, an extra peak is observed, indicated by the pound sign (#). (b) Barplot of the original repeat distance of the LPP, which is elongated in the CC-FTM. Data represents mean ±SD of four independent experiments. (c) Drawing of lamellar organization in the intercorneocyte space, where d indicates the length of the periodicity phase, adapted from van Smeden *et al*. [34].

To evaluate the formation and organization of the lipid matrix in the epidermal barrier, we first studied the lamellar organization of the lipids in the stratum corneum (SC). Based on the unique X-ray diffraction pattern of the lipid lamellae, the identification and characterization of the lamellar phases was performed. In the obtained peak profile, the first, second and third orders of diffraction of the LPP are identified (Fig 3A). The repeat distance of the LPP is determined by the peak position of the first, second and third order of diffraction, which revealed elongation in CC-FTMs (Fig 3B). In addition to the LPP, the presence of crystalline cholesterol is also detected. Furthermore, an additional peak with unidentified origin is detected in the diffraction profile of the FTM, which is absent in CC-FTMs.

### Lipid content and ceramide chain length distribution in CC-FTMs

**Fig 4:**
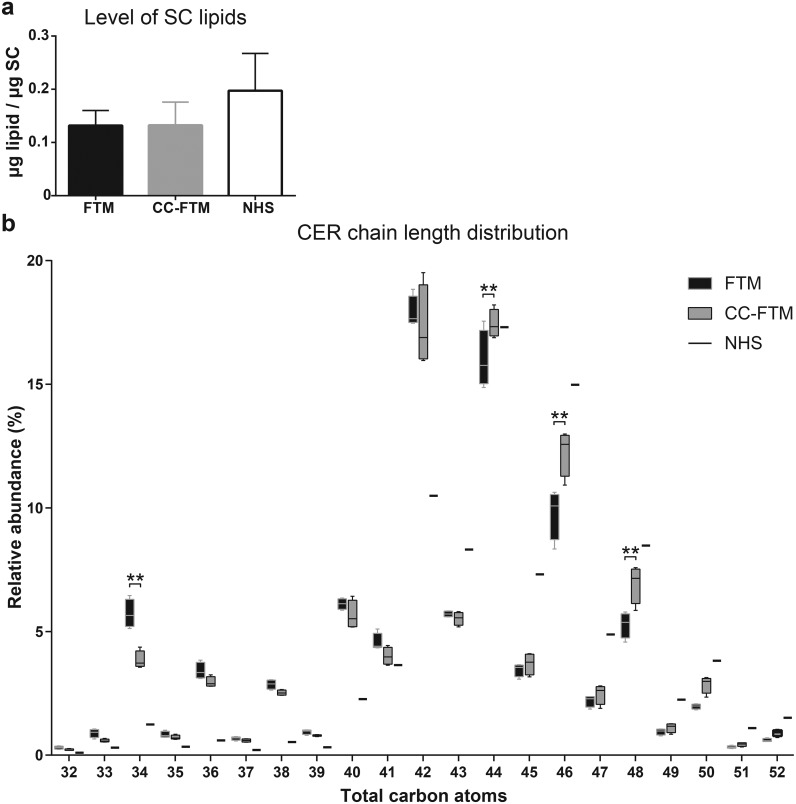
Lipid content and relative abundance of ceramides with specific carbon chain length. (a) The level of lipids in the SC of the FTM and CC-FTM is equal. (b) Box and whisker plot of CERs as function of increasing number of C-atoms in the total hydrocarbon chain, with indicated benchmark values of NHS. In CC-FTMs, a decreased abundance of C34 CERs is indicated, whereas there is an increase in C44, C46 and C48 CERs. Whiskers indicate the 95% confidence interval. Data is obtained from four independent experiments.

After the observation that modulation of the dermal matrix positively influenced the SC lamellar organization, the total lipid content of the SC and the ceramide (CER) composition were determined. The level of total lipids in the SC of both models remained equal (Fig 4A), which suggests that the addition of chitosan to the dermal matrix does not affect the quantity of total lipids in the SC.

Subsequently, the composition of the ceramides (CERs) of both FTM types was investigated, to provide more insight in the barrier formation. To this end, the lipids were extracted from the isolated SC, separated by mass and polarity using a detailed LC-MS approach to determine the CER profiles. First, the twelve well known ceramide subclasses were detected in both FTM types and in a mixture of lipid extracts from NHS. Furthermore, several unidentifiable species were detected (S2 Fig), although due to their unknown nature these are not analysed in detail. The overall CER chain length distribution is compared for both FTM types and plotted separately for ceramides (Fig 4B) and ω-esterified ceramides (EO-CERs) (S3 Fig) together with the benchmark values of NHS. When focusing on the differences between the FTM types, a reduction in CERs with a total of 34 carbon atoms in the two chains (C34 CERs) is detected in the CC-FTM. Furthermore, an increase in the level of C44, C46 and C48 CERs is observed. These alterations improve the resemblance of the CER chain length distribution in CC-FTMs to NHS. To obtain more detailed knowledge about which CER subclasses mainly contribute to these differences, the CER subclass profiles of these specific CERs were determined (S4 Fig). This reveals that the decreased relative abundance of C34 CERs is attributed to the subclasses NS and AS. The increased relative abundance of C44, C46 and C48 CERs is mainly attributed to the CER subclasses NdS, NP and NH. When focusing on the EO-CERs chain length distribution, a reduction in the level of C66, C68 and C70 EO-CERs, is observed in CC-FTMs. These observations indicate that modulation of the dermal matrix affects the CER chain length distribution.

### Profile of the ceramide subclasses in CC-FTMs

**Fig 5:**
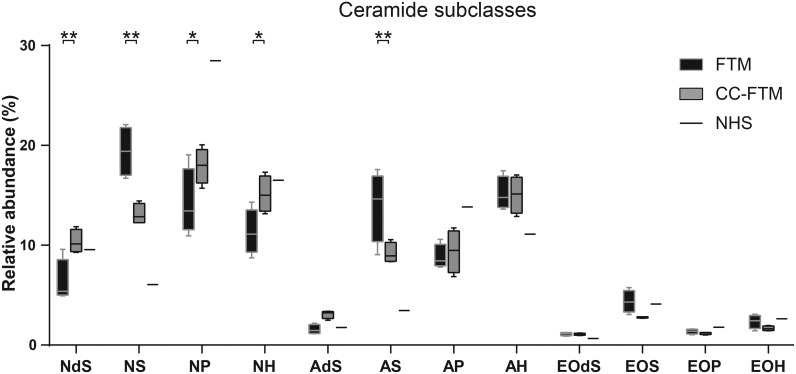
Ceramide subclass profile in FTMs and CC-FTMs. Box and whisker plot of CER subclasses with indicated benchmark values of NHS. Significant differences in relative abundance are indicated, which are the reduction in NS and AS and an increase of NdS, NP and NH in SC of CC-FTMs compared to FTMs. Whiskers indicate the 95% confidence interval. Data is obtained from four independent experiments.

Besides the importance to mimic the CER chain length distribution, the overall CER subclass profile should also resemble that of NHS. Based on the separation and detection of the CERs with the LC-MS analysis, the relative abundance of each CER subclass in the SC was determined. The relative abundance of each CER subclass is plotted for both FTM types (Fig 5). Several differences in the relative abundance of the CER subclasses are observed. The most significant changes are the reduction in the level of the NS and AS subclasses in the CC-FTM. Compensatory, an increased level of NdS, NH and notably NP in the CC-FTM is observed. The relative abundance of AdS, AP, AH and EO-CERs remained equal in both FTM types. The significant alterations in the CER subclass profile induces that the profile of NHS is more mimicked by the CC-FTM than the FTM.

### Inside-out barrier functionality of the CC-FTMs

**Fig 6:**
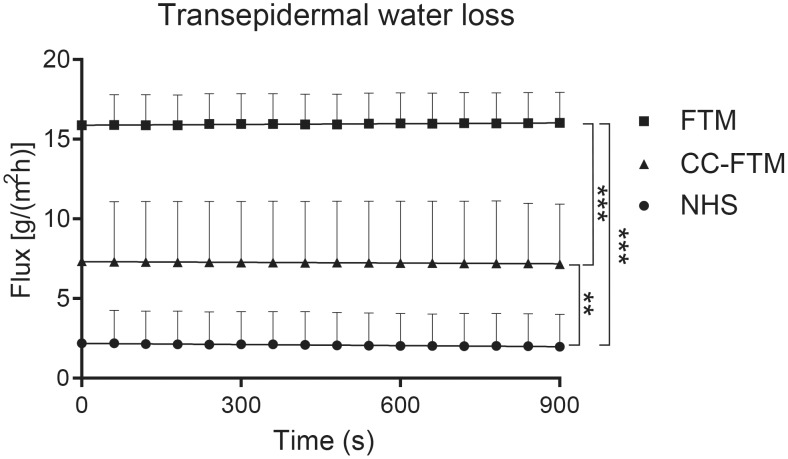
Transepidermal water loss of NHS, FTMs and CC-FTMs. TEWL was measured over 900 second time period and plotted as mean values +SEM. Data is obtained from three independent experiments and three NHS samples.

Followed by the detailed examination of the SC lipid barrier organization and CER composition, the barrier functionality of the NHS, FTMs and CC-FTMs is determined. Stable measurements of the transepidermal water loss (TEWL) over time revealed a significant reduction of the TEWL in the CC-FTMs compared to the FTMs (Fig 6). The adjustment of the dermal matrix induced an improved inside-out barrier functionality based on compared TEWL values of NHS and both FTM types.

### Expression of major lipid processing enzymes

**Fig 7:**
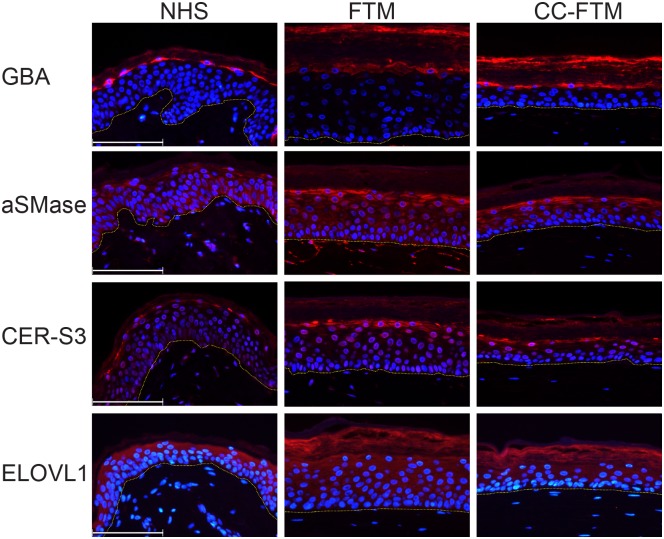
Expression of lipid processing enzymes in NHS, FTM and CC-FTM. Tissue sections of NHS, FTMs and CC-FTMs are analysed by immunofluorescence for the expression of lipid processing enzymes GBA, aSMase, CER-S3 and ELOVL1. Representative images show the localization (red) of GBA, aSMase and CER-S3 in the stratum granulosum, while the expression of ELOVL1 is more diffuse throughout the epidermis. Nuclei are stained blue using DAPI, the yellow dotted line indicates the dermal-epidermal junction. Scale bar represents 100μm.

The observed alterations in both CER chain length distribution and CER subclass profile could be a result of an altered expression of the major lipid processing enzymes. We therefore examined the expression GBA, aSMase, CER-S3 and ELOVL1 in NHS and both FTM types (Fig 7). The expression of GBA and aSMase, both critical in the final step of ceramide synthesis, is localized in the granular layer of the epidermis in both FTM types, similar to NHS. The restriction to a single granular layer of GBA in CC-FTMs mimics more closely the expression of GBA in NHS. In addition, the expression of aSMase in the CC-FTM is more localized towards the granular layer. The expression of CER-S3, important in the formation of ceramides through linkage of the sphingoid backbone to very long chain fatty acids, is restricted to the granular layer in both models and in NHS. Finally, the expression of ELOVL1, which plays a role in the elongation of FFA chains, is localized in all epidermal layers equally for NHS, FTM and CC-FTM.

## Discussion

Many properties of the native human skin (NHS) are mimicked in vitro in the range of existing HSEs. However, there is still an urgent need for HSEs that resemble the epidermal barrier properties very closely. In this work, CC-FTMs were developed through modulation of the dermal matrix by incorporation of the biopolymer chitosan. A major practical advantage of the collagen-chitosan dermal equivalent is the generation of it in a single fully hydrated preparation step, instead of a double preparation step for the FTMs. This novel FTM type is thoroughly analysed for epidermal morphogenesis and barrier formation and compared to the established FTM.

The epidermis of both FTM types is well-organized, based on the distinguishable presence of all four epidermal layers. In the CC-FTM, the epidermal thickness is reduced as well as the proliferation in the basal layer. However, the proliferation index (PI) of both FTM types is within the reported range of PIs for NHS [37], which is further confirmed by our findings. The epidermal differentiation program maintained regulated in both FTM types, although the granular layer in CC-FTM is reduced to approximately a single layer in CC-FTMs. The epidermal hyper-proliferation associated K16 is detected in FTMs and in several commercially available models [38], but is absent in NHS [39] and CC-FTMs, indicative for an improved epidermal homeostasis. Previously, it has been shown that the expression of K16 depends on the number of fibroblasts present in the dermal matrix [39], demonstrating a highly significant role for the fibroblasts and their microenvironment in the regulation of epidermal homeostasis and subsequent epidermal functionality.

The crucial role of the epidermal-dermal communication in both FTM types was analysed through expression of major BM proteins. The presence of the BM was detected in both FTM types, although the modulation of the dermal matrix attenuates the deposition of laminin 332 (L332). The major biological function of L332 is to facilitate epidermal attachment [40–42]. The reduced deposition of L332 in the CC-FTMs cannot be induced by an altered fibroblast distribution, based on the detection of the mesenchymal marker vimentin throughout the dermis of both FTM types. The reduced deposition of L332 could be explained by a functional overlap of L332 and chitosan, where both could act as substrate for keratinocyte adhesion molecules. This could reduce the necessity for the deposition of L332. This altered adhesion in turn could affect basal cell proliferation. Direct interaction of keratinocytes with a chitosan film has been observed before [24]. However, the direct attachment of keratinocytes to chitosan in the collagen-chitosan matrix should be investigated in detail in future studies.

The functionality of the epidermal barrier in the SC highly depends on terminally differentiated keratinocytes in concert with the SC extracellular lipid matrix. Expression of loricrin as major cornified envelope protein is similar in both FTM types, as well as the level of total lipids in the SC. The number of corneocyte layers in the SC of both FTM types is equal and comparable but not equal as in NHS [43].

Next, the lamellar organization of the SC lipids was determined revealing an increased repeat distance of the LPP in CC-FTMs. Since the repeat distance of the LPP in NHS is approximately 13 nm [44], this indicates that the lamellar organization of CC-FTMs resembles that of NHS to a higher extent but still not completely. Moreover, the additional unknown lamellar phase in FTMs, possibly explained by phase separated CERs, is not present in CC-FTMs and NHS [8]. The SPP of approximately 6 nm has been identified in NHS, but has not yet been detected in any human skin model [5, 44].

To possibly explain the altered lipid organization and to gain more insight in the lipid barrier formation, the CER composition was analysed. First, the CER chain length distribution was assessed, revealing a wide range in CER carbon chain lengths in both FTM types. This demonstrates once more that the synthesis of the high variable CERs entities in FTMs is functional, which is a major characteristic of NHS. When comparing both FTM types, there is a decreased relative abundance of C34 CERs in CC-FTMs detected. Compensatory, an increment in the relative abundance of C44, C46 and C48 CERs in CC-FTM was observed. Next, a comparison is made with the CER chain length distribution of NHS, based on the benchmark values reported here and values described in literature [3, 35]. The reduced relative abundance of C34 CERs and the increased relative abundance of C44, C46 and C48 CERs in the CC-FTM compared to FTM resemble the chain length distribution of NHS more closely. An increase in the chain length of CERs has been correlated with a decrease in transepidermal water loss (TEWL) and thus an increased skin barrier [45]. This indicates that the CER composition influences the functionality of the epidermal barrier and that the barrier in CC-FTMs is superior to FTMs.

Second, the CER subclass profiles of both FTM types are compared to each other. In the CC-FTM, the CER subclass NP is increased to a level where this subclass is relatively the most abundant. Also CER NdS and NH are relatively more abundant. In contrast, the relative abundance of both AS and NS are reduced in the SC of CC-FTMs. The presence of EO-CERs is lower than reported before in HSEs [7], which is ascribed to the utilized corrections. In this study the level of EO-CERs are underestimated, since no chain length corrections have been performed. However, this does not affect the conclusions of our present study. To evaluate to which order the CER subclass profiles resembles that of NHS, we compared the FTM and CC-FTM CER subclass profiles to the CER subclass profile of NHS [45]. The CER subclass profile in NHS has been studied extensively before with increased precision based on technical progress [3, 35, 46–49]. In NHS, the CER subclass NP is most abundant, which is most similar to the CC-FTM. In addition, the decreased relative abundance of CER subclasses AS and NS, as in the CC-FTMs, is more close to the CER subclass profile of NHS [3, 35, 46–49]. Altogether, the CER composition in the SC of CC-FTMs resembles more that of NHS, based on the CER chain length distribution and CER subclass profile.

Several interesting observations are made when comparing the obtained results from both FTM types to the epidermal barrier formation of skin in an unbalanced or diseased state. This is performed through comparing with atopic dermatitis (AD), a skin condition which is studied extensively regarding epidermal barrier formation [45, 50]. Although the aetiology is different, several similarities are observed. The epidermis of AD, most profound in lesional AD, as well as the FTMs, shows an elevated expression of K16 and increased proliferation [51], both indicative for an epidermis which is not in homeostasis. Furthermore, the increased relative abundance of CER subclasses AS, NS and the C34 CERs are observed in AD and FTMs, but are more normalized in the CC-FTMs. Therefore the chitosan modulated dermal matrix seems to be highly beneficial for the epidermal homeostasis, including the epidermal barrier formation.

This is further emphasized by the barrier functionality assay. TEWL measurements are widely applied and regarded indicative for the skin barrier integrity [34, 36, 52]. In the CC-FTMs the TEWL is significantly reduced, which may be a result of the altered SC lipid composition. This underlines the importance of the SC lipid composition in the formation of a competent barrier.

Finally, the expression of CER processing enzymes is studied, to evaluate if the altered CER composition is a direct effect of differently expressed enzymes. This is determined through analysis of the expression of GBA and aSMase. The localization of GBA at the interface of the stratum granulosum and stratum corneum is in agreement with the site where the conversion of CER precursors occurs. The intensity and restriction to a single granular layer of GBA in CC-FTMs mimics more the NHS. The localization of aSMase is not affected by the modulated dermal matrix. Although the level of AS and NS is reduced in CC-FTMs, this cannot be linked to an altered expression by aSMase, which processes the sphingomyelin into the CER subclasses AS and NS [53, 54]. However, the functionality of both enzymes can still be affected. The expression and localization of CER-S3 and ELOVL1 is highly similar in both FTM types and NHS [55], indicating that the major lipid metabolism pathways are unaffected in vitro. The expression of major lipid processing enzymes in the CC-FTM is highly comparable to the FTMs, although the expression in the granular layer is more restricted to a single layer in CC-FTMs and NHS, which could be beneficial for the functionality of these enzymes.

## Conclusion

The epidermal barrier properties of human skin models needs to resemble the NHS barrier properties to a higher extent, in order to fully mimic the NHS *in vitro*. In this work, the development of CC-FTMs is described, through modulation of the dermal matrix by the biopolymer chitosan. The CC-FTM resembles NHS to a higher extent regarding epidermal morphogenesis. The formation of the epidermal barrier, based on the organization and composition of the SC lipids and the inside-out barrier functionality, mimics the NHS more closely, although not entirely. The novel CC-FTMs are a promising tool to be used in epidermal barrier related studies.

## Supporting information

S1 Table Specification of antibodies used for immunohistochemical and immunofluorescence staining. (DOCX)

S1 Fig Overview of molecular structure of general ceramides and twelve ceramide subclasses. Ceramides contain a fatty acid chain (grey) amide linked to a sphingosine chain (blue). Red parts indicate positions with variable architectures. Red arrows indicate chain length variability. Nomenclature and structure of the twelve well-known ceramide subclasses is provided in the table. Reprinted from Janssens and Smeden *et al*. [45] under a CC BY license, with permission from JLR/ASBMB, original copyright 2012.
(TIF)

S2 Fig Two dimensional LC-MS plot of the ceramides isolated from the stratum corneum. The location and nomenclature of the twelve well-known ceramide subclasses are provided in two representative plots. Dashed lines indicate unknown lipid entities. Data is obtained from four independent experiments.
(TIF)

S3 Fig Relative abundance of EO-CERs with specific carbon chain length in FTMs and CC-FTMs. Box and whisker plot of EO-CERs with different number of C-atoms and indicated benchmark values of NHS. In CC-FTMs, a decrease of C66, C68 and C70 EO-CERs is detected. Whiskers indicate the 95% confidence interval. Data is obtained from four independent experiments.
(TIF)

S4 Fig Relative abundance of C34 and C44-48 CERs per subclass. Box and whisker plots of CER subclasses from only the (a) C34 CERs, (b) C44 CERs, (c) C46 CERs and (d) C48 CERs. Benchmark values of NHS are indicated for each subclass. The alteration in relative abundance of these groups is described to only some CER subclasses. These are NS and AS in C34 CERs, NdS, NS, NP, NH and AS in C44 CERs, NdS, NP and NH in C46 CERs and NdS, NP and NH in C48 CERs. Whiskers indicate the 95% confidence interval, *indicates p<0.05. Data is obtained from four independent experiments.
(TIF)
