# Supplementary material for: Improved epidermal barrier formation in human skin models by chitosan modulated dermal matrices
Source: PLoS One. 2017 Mar 23;12(3):e0174478. doi: 10.1371/journal.pone.0174478 (PMC5363943; doi:10.1371/journal.pone.0174478)
Supplement: S1 Table — (DOCX) [file pone.0174478.s001.docx]

| **Immunohistochemistry** | **Origin** | **Clone** | **Dilution** | **Secondary antibody** | **Manufacturer** |
| --- | --- | --- | --- | --- | --- |
| ***Primary antibody*** |  | | |  |  |
| Ki67 | Mouse | MIB1 | 1:100 | A | DAKO, Denmark |
| cytokeratin 10 | Mouse | DE-K10 | 1:50 | A | Labvision/Neomarkers, USA |
| cytokeratin 16 | Mouse | LL025 | 1:50 | A | Serotec, UK |
| ***Secondary antibody*** |  | | | | |
| Biotinylated Goat anti-mouse (A) | Goat |  | 1:200 |  | Southern Biotechnology |
| **Immunofluorescence** | **Origin** | **Clone** | **Dilution** | **Secondary antibody** | **Manufacturer** |
| ***Primary antibody*** |  |  | | | |
| Loricrin | Rabbit | AF62 | 1:1000 | B | Covance, The Netherlands |
| Vimentin | Mouse | V6630 | 1:50 | C | Sigma-aldrich, The Netherlands |
| Collagen type IV | Mouse | 24.12.8 (PHM-12) | 1:75 | C | Chemicon, Australia |
| Laminin 332 | Mouse | BM165 | 1:75 | C | Dr. M. Aumailley, Germany |
| GBA | Rabbit | EPR5142 | 1:150 | D | Abcam, UK |
| aSMASE | Rabbit | Polyclonal | 1:1000 | D | Abcam, UK |
| CER-S3 | Rabbit | Polyclonal | 1:75 | D | Sigma-aldrich, The Netherlands |
| ELOVL1 | Rabbit | Polyclonal | 1:300 | D | Santa Cruz, USA |
| ***Secondary antibody*** |  | | | | |
| Cy3-conjugated anti-rabbit (B) | Goat |  | 1:500 |  | Jackson immunoresearch Laboratory, UK |
| Cy3-conjugated anti-mouse (C) | Goat |  | 1:1000 |  | Jackson immunoresearch  Laboratory, UK |
| Rhodamine Red-X anti-rabbit (D) | Goat |  | 1:300 |  | Jackson immunoresearch Laboratory, UK |
