# Supplementary figures and images for: Improved epidermal barrier formation in human skin models by chitosan modulated dermal matrices
Source: PLoS One. 2017 Mar 23;12(3):e0174478. doi: 10.1371/journal.pone.0174478 (PMC5363943; doi:10.1371/journal.pone.0174478)

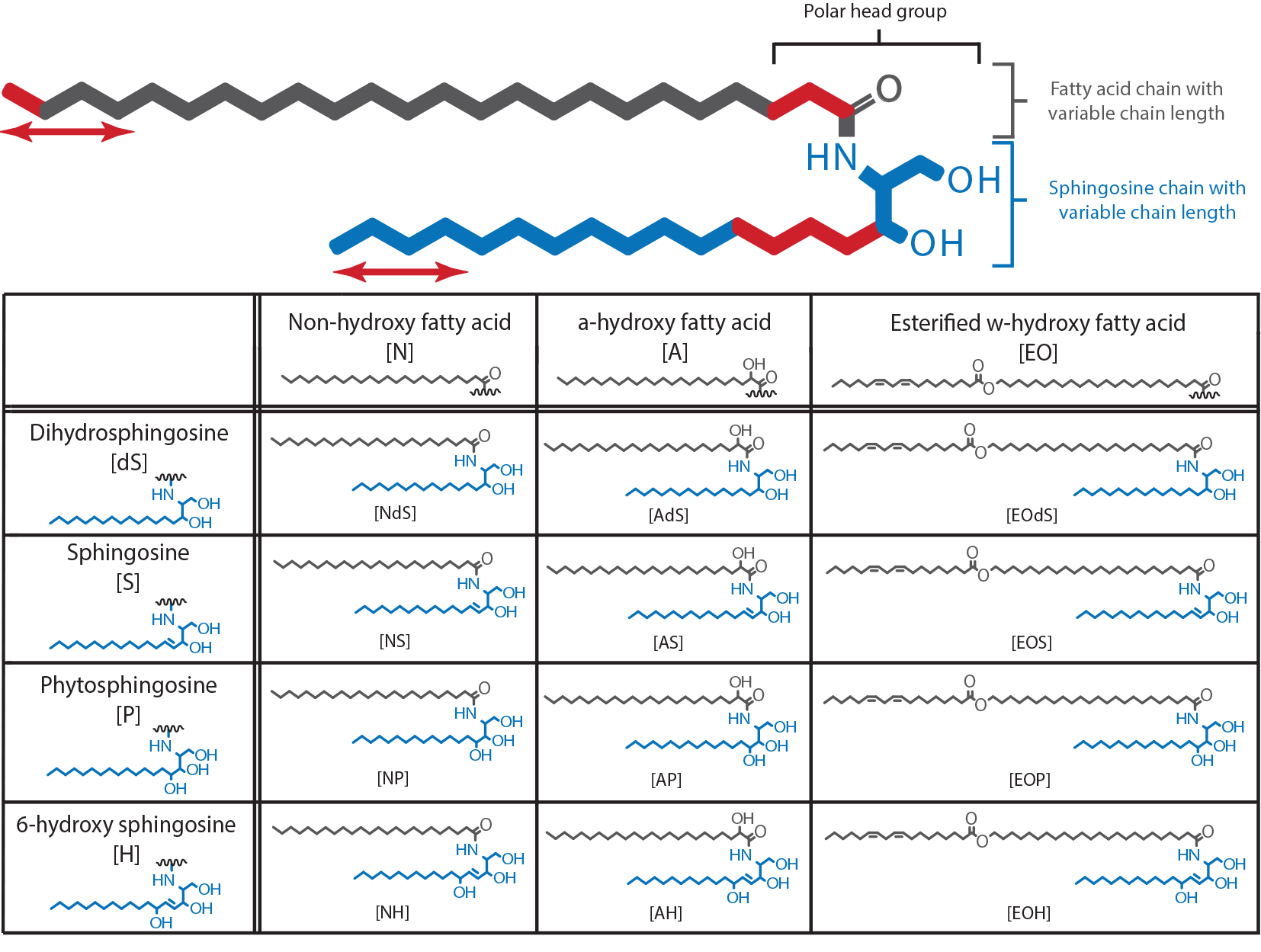

Supplement: S1 Fig — Ceramides contain a fatty acid chain (grey) amide linked to a sphingosine chain (blue). Red parts indicate positions with variable architectures. Red arrows indicate chain length variability. Nomenclature and structure of the twelve well-known ceramide subclasses is provided in the table. Reprinted from Janssens and Smeden et al. [45] under a CC BY license, with permission from JLR/ASBMB, original copyright 2012. (TIF) [file pone.0174478.s002.tif]

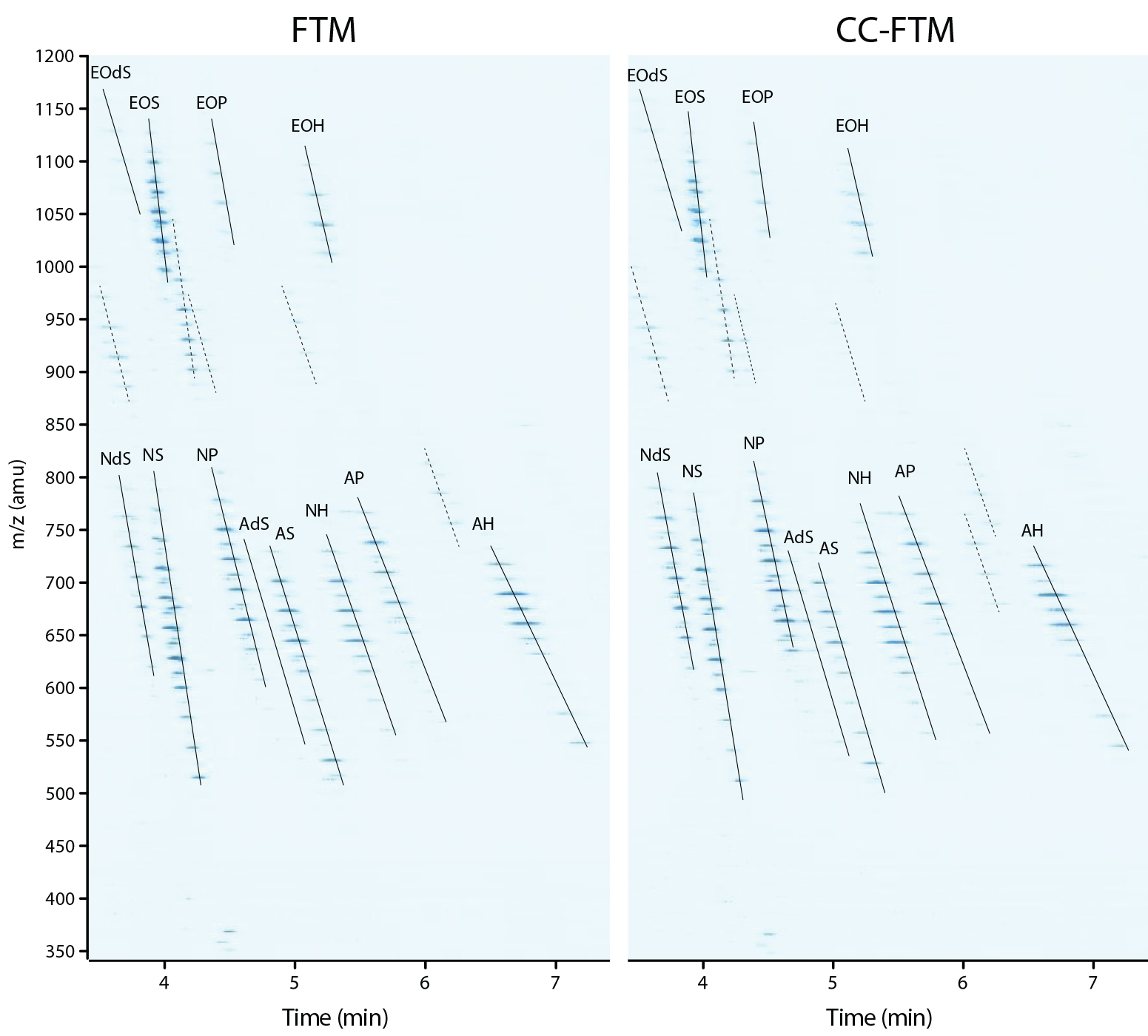

Supplement: S2 Fig — The location and nomenclature of the twelve well-known ceramide subclasses are provided in two representative plots. Dashed lines indicate unknown lipid entities. Data is obtained from four independent experiments. (TIF) [file pone.0174478.s003.tif]

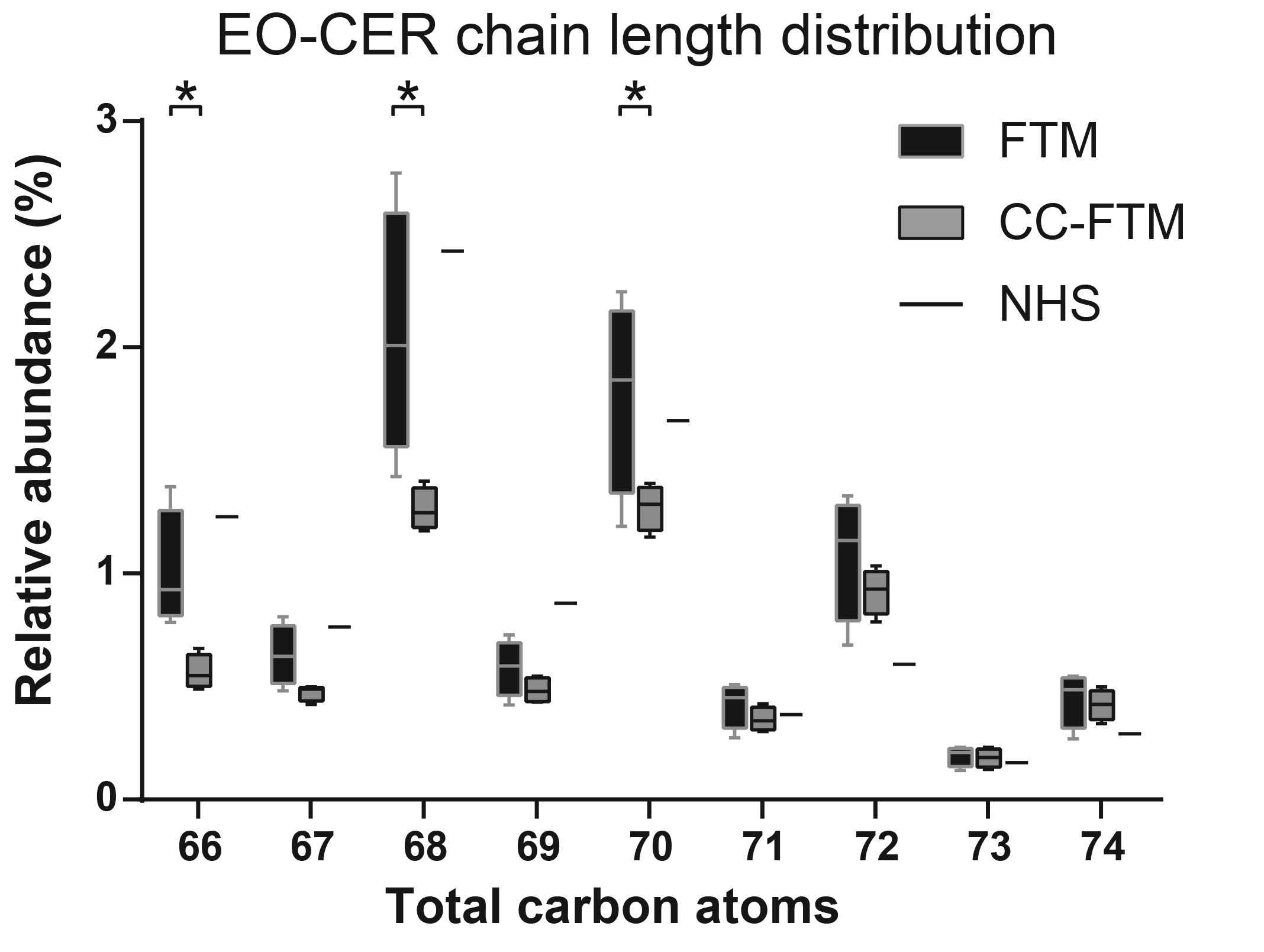

Supplement: S3 Fig — Box and whisker plot of EO-CERs with different number of C-atoms and indicated benchmark values of NHS. In CC-FTMs, a decrease of C66, C68 and C70 EO-CERs is detected. Whiskers indicate the 95% confidence interval. Data is obtained from four independent experiments. (TIF) [file pone.0174478.s004.tif]

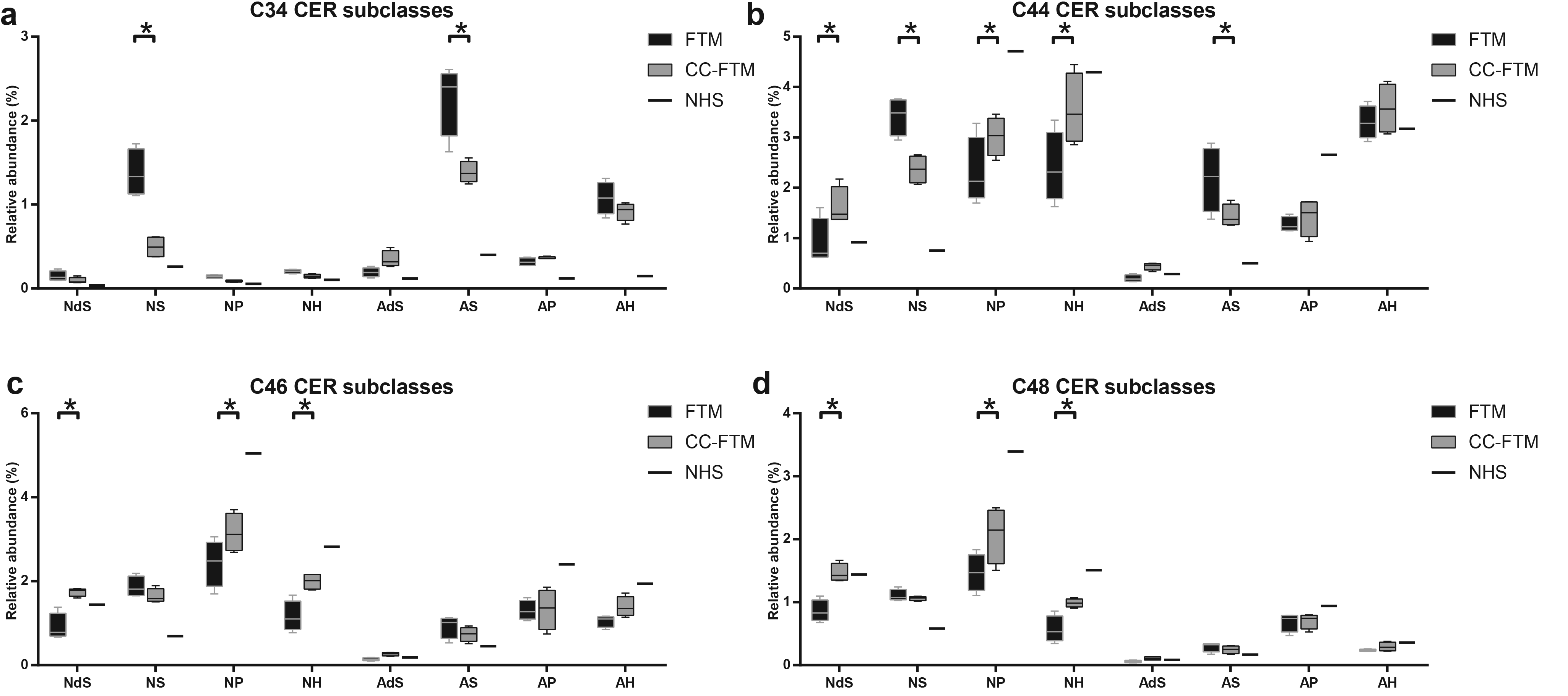

Supplement: S4 Fig — Box and whisker plots of CER subclasses from only the (a) C34 CERs, (b) C44 CERs, (c) C46 CERs and (d) C48 CERs. Benchmark values of NHS are indicated for each subclass. The alteration in relative abundance of these groups is described to only some CER subclasses. These are NS and AS in C34 CERs, NdS, NS, NP, NH and AS in C44 CERs, NdS, NP and NH in C46 CERs and NdS, NP and NH in C48 CERs. Whiskers indicate the 95% confidence interval, *indicates p<0.05. Data is obtained from four independent experiments. (TIF) [file pone.0174478.s005.tif]
